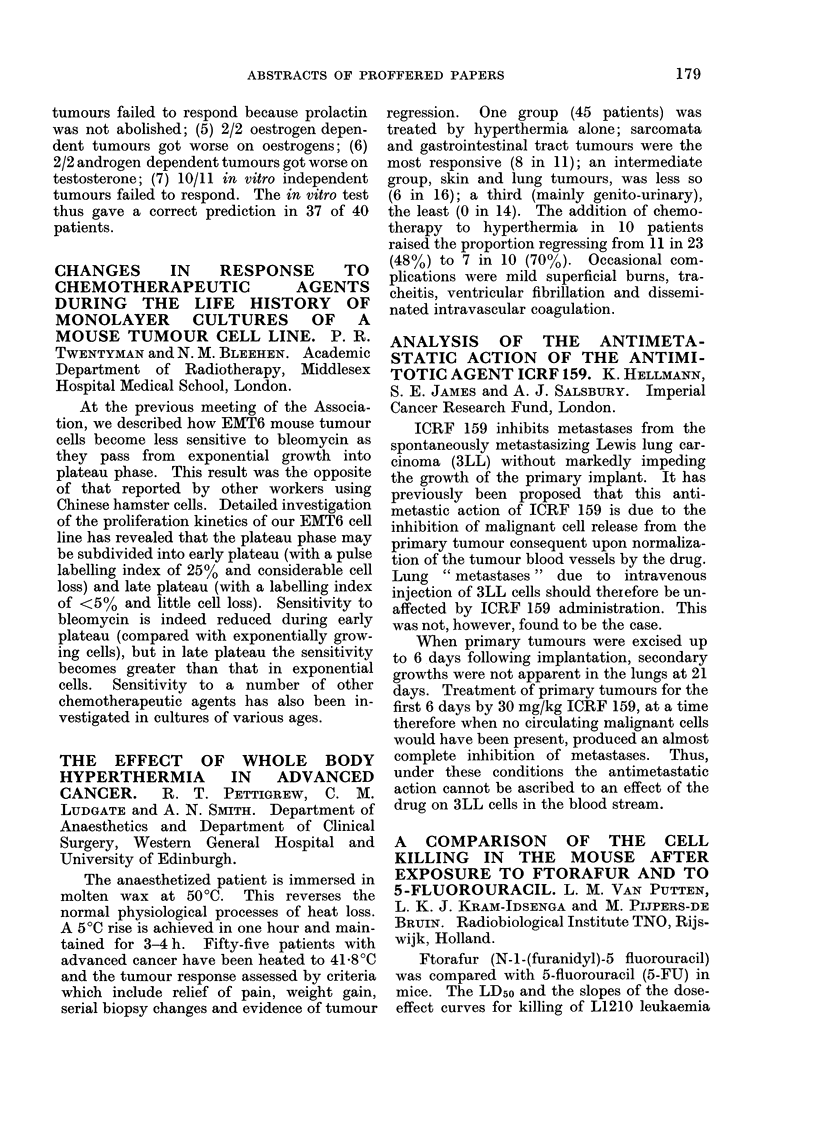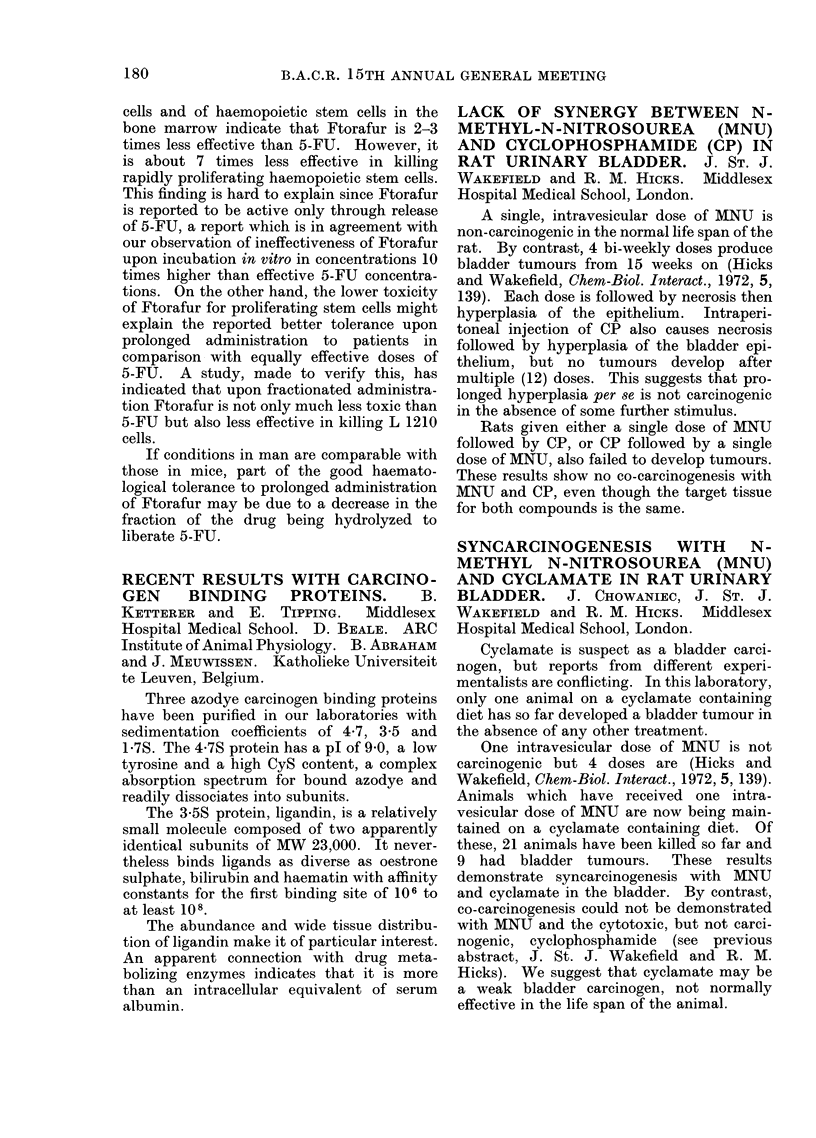# Proceedings: A comparison of the cell killing in the mouse after exposure to ftorafur and to 5-fluorouracil.

**DOI:** 10.1038/bjc.1974.154

**Published:** 1974-08

**Authors:** L. M. Van Putten, L. K. Kram-Idsenga, M. Pijpers-de Bruin


### A COMPARISON OF THE CELL KILLING IN THE MOUSE AFTER EXPOSURE TO FTORAFUR AND TO 5-FLUOROURACIL.

L. M. Van Putten, L. K. J. Kram-Idsenga and M. Pijpers-de Bruin. Radiobiological Institute TNO, Rijswijk, Holland.

Ftorafur (N-1-(furanidyl)-5 fluorouracil) was compared with 5-fluorouracil (5-FU) in mice. The $LD_{50}$ and the slopes of the dose-effect curves for killing of L1210 leukaemia cells and of haemopoietic stem cells in the bone marrow indicate that Ftorafur is 2–3 times less effective than 5-FU. However, it is about 7 times less effective in killing rapidly proliferating haemopoietic stem cells. This finding is hard to explain since Ftorafur is reported to be active only through release of 5-FU, a report which is in agreement with our observation of ineffectiveness of Ftorafur upon incubation *in vitro* in concentrations 10 times higher than effective 5-FU concentrations. On the other hand, the lower toxicity of Ftorafur for proliferating stem cells might explain the reported better tolerance upon prolonged administration to patients in comparison with equally effective doses of 5-FU. A study, made to verify this, has indicated that upon fractionated administration Ftorafur is not only much less toxic than 5-FU but also less effective in killing L 1210 cells.

If conditions in man are comparable with those in mice, part of the good haematological tolerance to prolonged administration of Ftorafur may be due to a decrease in the fraction of the drug being hydrolyzed to liberate 5-FU.